# Effect of Quercetin on Dexamethasone-Induced C2C12 Skeletal Muscle Cell Injury

**DOI:** 10.3390/molecules25143267

**Published:** 2020-07-17

**Authors:** Chun Chen, Jai-Sing Yang, Chi-Cheng Lu, Yu-Jen Chiu, Hung-Che Chen, Mei-Ing Chung, Yu-Tse Wu, Fu-An Chen

**Affiliations:** 1 School of Pharmacy, College of Pharmacy, Kaohsiung Medical University, Kaohsiung City 807, Taiwan; u106830006@kmu.edu.tw (C.C.); meinch@kmu.edu.tw (M.-I.C.); 2 Department of Medical Research, China Medical University Hospital, China Medical University, Taichung 404, Taiwan; jaisingyang@gmail.com; 3 Department of Sport Performance, National Taiwan University of Sport, Taichung 404, Taiwan; a722353@ntupes.edu.tw; 4 Division of Plastic and Reconstructive Surgery, Department of Surgery, Taipei Veterans General Hospital, Taipei 112, Taiwan; chiou70202@gmail.com; 5 Department of Pharmacy and Master Program, Tajen University, Pingtung 907, Taiwan; tetsu21@tajen.edu.tw; 6 Drug Development and Value Creation Research Center, Kaohsiung Medical University, Kaohsiung 807, Taiwan

**Keywords:** apoptosis, antioxidant, C2C12 skeletal muscle cells, dexamethasone, mitochondrial membrane potential (ΔΨm), quercetin

## Abstract

Glucocorticoids are widely used anti-inflammatory drugs in clinical settings. However, they can induce skeletal muscle atrophy by reducing fiber cross-sectional area and myofibrillar protein content. Studies have proven that antioxidants can improve glucocorticoid-induced skeletal muscle atrophy. Quercetin is a potent antioxidant flavonoid widely distributed in fruits and vegetables and has shown protective effects against dexamethasone-induced skeletal muscle atrophy. In this study, we demonstrated that dexamethasone significantly inhibited cell growth and induced cell apoptosis by stimulating hydroxyl free radical production in C2C12 skeletal muscle cells. Our results evidenced that quercetin increased C2C12 skeletal cell viability and exerted antiapoptotic effects on dexamethasone-treated C2C12 cells by regulating mitochondrial membrane potential (ΔΨm) and reducing oxidative species. Quercetin can protect against dexamethasone-induced muscle atrophy by regulating the Bax/Bcl-2 ratio at the protein level and abnormal ΔΨm, which leads to the suppression of apoptosis.

## 1. Introduction

Glucocorticoids are widely prescribed for treating inflammatory disorders, asthma, allergic rhinitis, ulcerative colitis, ophthalmic, dermatological, neurological, and autoimmune diseases, and even hematological cancers [1,2,3,4]. They have been proposed to be an essential part of first-line anti-inflammatory treatment [5]. However, glucocorticoids can downregulate the rate of skeletal muscle protein synthesis by increasing the rate of protein breakdown [6,7]. Consequently, they induce myopathy, which is a major clinical problem in chronic corticosteroid treatment [8]. Myostatin, a member of the transforming growth factor beta (TGF-β) superfamily, is a negative muscle cell growth regulator and downregulates the proliferation and differentiation of satellite cells and muscle hypertrophy by activating Smad2 and dephosphorylates Akt pathways [9,10]. Glucocorticoid-induced skeletal muscle atrophy is associated with the upregulation of myostatin gene expression [11], indicating that myostatin plays a crucial role in dexamethasone-induced muscle atrophy [12]. Myostatin is a contributor to mitochondrial metabolic alterations and mitochondria-dependent apoptosis [13,14]. Moreover, it induces reactive oxygen species (ROS) production, impairs Ca^2+^ handling, and releases mitochondria-specific proteolytic activators [15,16].

Skeletal muscle apoptosis or programmed cell death is a major index of sarcopenia [17,18,19,20,21]. Skeletal muscle apoptosis has various mechanisms, one of which involves mitochondrial apoptotic signaling regulation via its inhibitory effects on Akt phosphorylation. In addition, Bad serine 136 phosphorylation is a result of the environment not being appropriate for cell proliferation; it induces the processing of apoptosis via Bad activation [22,23]. Thus, inhibition of Bad signaling reduces apoptosis [17,24]. Compounds with antioxidant properties have demonstrated the ability to delay muscle atrophy [25].

In foods, onions are major sources of quercetin (3,3′,4′,5,7′-pentahydroxyflavone) and its contents varied extensively from 16.10 to 103.93 mg/g in dry-weight [26,27,28]. Furthermore, commercial health food is also available for providing alternative sources of quercetin (500 mg/Capsule). In general, the range of typical dosages is 500~1000 mg per day. Quercetin is a natural and powerful antioxidant chemopreventive agent; it provides protective effects against glutathione (GSH), enzymatic activity, signal transduction pathways, and ROS-induced oxidation [29]. In human aortic endothelial cells, quercetin affects glutathione levels and redox ratio regulation not through oxidation but through the formation and cellular export of quercetin-glutathione conjugates and upregulation of glutamate-cysteine ligase extracellular quercetin-glutathione conjugates [30]. Furthermore, quercetin alters hepatic GSH metabolism by modulating GSH metabolic enzyme activities via p38, ERK1/2 MAPKs, and Nrf2 pathways in rats [31]. Studies have demonstrated that polyphenols could reduce dexamethasone-induced muscle atrophy and downregulate muscle atrophy-related proteins such as ubiquitin ligase atrogin-1 and MuRF-1 [32,33].

Dexamethasone induces mitochondrial malfunction via *Drp1, PGC-1*, *NRF1*, and *TFam* downregulation, *Mfn2* upregulation, respectively [34]. Those mitochondrial biogenesis and dynamics factors cause intracellular ATP deprivation and robust AMPK activation, which further activates the FOXO3/Atrogenes pathway [35]. However, whether quercetin can improve the dexamethasone-induced ΔΨm imbalance and provide anti-apoptosis activity remains unclear. In our study, dexamethasone stimulated ROS production in C2C12 cells, increased Bax protein levels, and attenuated Bcl-2 protein expression in dexamethasone-treated C2C12 cells. Dexamethasone suppressed the viability of C2C12 cells by inhibiting cell proliferation and triggering mitochondria-mediated apoptotic cell death. We demonstrated that quercetin prevented disuse-related muscle atrophy it provided insight into problems of dexamethasone-induced peroxidation on skeletal muscle injury via its regulation of ΔΨm.

## 2. Results

### 2.1. Quercetin Reduces Growth Inhibition and Triggers Morphological Changes in Dexamethasone-Treated C2C12 Myotube Cells

**Figure 1:**
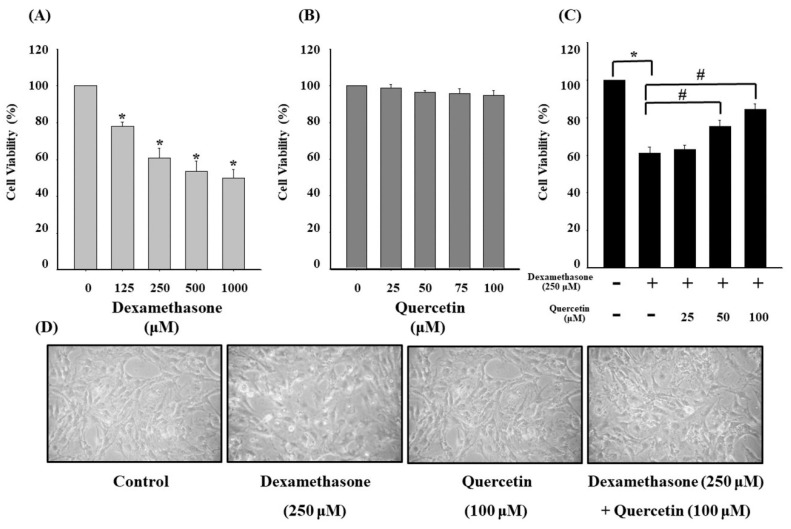
(**A**) Cell viability of C2C12 myotube cells in the presence of 0, 125, 250, 500, and 1000 μM dexamethasone for 4 h. (**B**) Cell viability of C2C12 myotube cells in the presence of 0, 25, 50, 75, and 100 μM quercetin for 24 h. (**C**) Cell viability of C2C12 myotube cells treated with 25, 50, and 100 μM quercetin for 24 h and cotreated with 250 μM dexamethasone for 4 h. (**D**) Morphological alterations of C2C12 myotube cells in the presence of 100 μM quercetin for 24 h and cotreated with 250 μM dexamethasone for 4 h. Values represent the mean ± SE (n = 8). Significant differences were determined using Dunnett’s test (* *p* < 0.05, compared with the untreated group; # *p* < 0.05, compared with the dexamethasone-treated group).

The effects of quercetin and dexamethasone on C2C12 myotube cell viability were respectively measured at various concentrations of dexamethasone (0, 125, 250, 500, and 1000 μM) for 4 h and quercetin (0, 25, 50, 75, and 100 μM) for 24 h, and the results are shown in Figure 1. Dexamethasone-treated groups exhibited a significant decrease in the cell viability of C2C12 myotube cells in a concentration-dependent manner (Figure 1A), whereas quercetin-treated groups exhibited no significant effect on cell viability at all tested concentrations (Figure 1B). Furthermore, quercetin significantly increased the cell viability of dexamethasone-treated C2C12 myotube cells in a concentration-dependent manner (Figure 1C). In addition, a morphological assay revealed that dexamethasone-treated C2C12 myotube cells detached from the surface of the plate and experienced cell membrane shrinkage. Quercetin treatment was evidenced to reduce cell membrane shrinkage in the morphology assays. Morphological alterations of C2C12 myotube cells were treated in the presence of 100 μM quercetin for 24 h and 250 μM dexamethasone for 4 h, respectively (Figure 1D).

The balance of protein turnover determines muscle mass which allows adapting to different pathophysiological conditions [36,37]. Muscle atrophy is characterized by the shrinkage of muscular tissues or organs caused by cell atrophy, resulting from the imbalance of muscle cells, organelles, cytoplasm, and proteins, which form two major cell proteolytic systems, namely, the ubiquitin–proteasome machinery and autophagy–lysosome machinery in muscle [36,38,39,40,41,42]. Quercetin reversed the dexamethasone-induced muscle atrophy and thus prevented myotube cell shrinkage.

### 2.2. Dexamethasone-Induced Loss of ΔΨm and the Elicited Caspase-Dependent Apoptosis in C2C12 Myotube Cells

**Figure 2:**
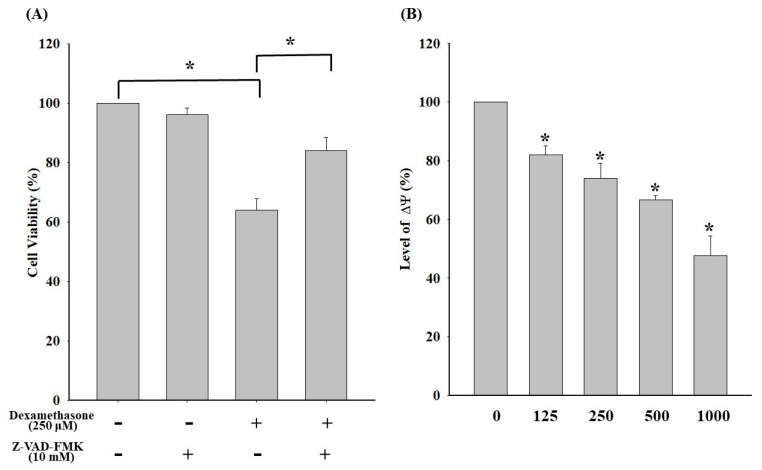
(**A**) Effect of the pan-caspase inhibitor Z-VAD-FMK on apoptosis in dexamethasone-treaded C2C12 myotube cells. Before dexamethasone treatment, the cells were pretreated with or without 10 μM Z-VAD-FMK for 24 h. (**B**) Effects of dexamethasone on ΔΨm in C2C12 myotube cells. Cells were incubated with 0, 125, 250, 500, and 1000 μM dexamethasone for 24 h. Values represent the mean ± SE (n = 8). Significant differences were determined using Dunnett’s test (* *p* < 0.05, compared with the untreated group).

To investigate the mechanism underlying dexamethasone-induced apoptosis in C2C12 cells, dexamethasone-induced C2C12 apoptosis associated with the caspase cascade signaling pathway was conducted. As shown in Figure 2A, the addition of Z-VAD-FMK (a pan-caspase inhibitor) significantly increased the cell viability of C2C12 cells compared with that of the group without the inhibitor. The results evidenced a major mechanism underlying the dexamethasone-induced apoptosis of C2C12 myotube cells associated with the caspase cascade pathway. In addition, ΔΨm levels were detected using a flow cytometric assay to further investigate the associated upstream signaling molecular pathways of dexamethasone-induced apoptosis. As a result, a trend of concentration-dependent ΔΨm decrease was observed in C2C12 myotube cells, indicating that dexamethasone-induced apoptosis was associated with ΔΨm imbalance (Figure 2B).

### 2.3. Effects of Quercetin on ROS Production and the Apoptotic Situation in Dexamethasone-Treated C2C12 Myotube Cells

**Figure 3:**
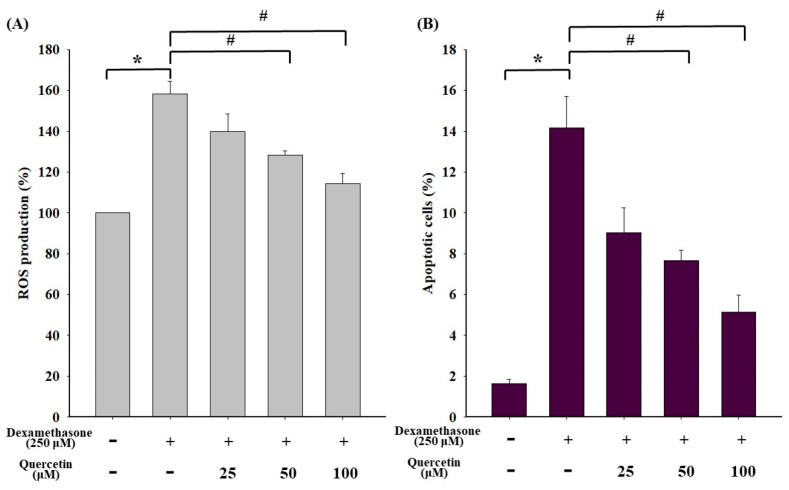
(**A**) Effect of dexamethasone on ROS production in C2C12 myotube cells. Cells were cotreated with 0, 25, 50, and 100 μM quercetin in the presence of 250 μM dexamethasone. (**B**) Effect of dexamethasone on apoptosis in C2C12 myotube cells. Cells were cotreated with 0, 25, 50, and 100 μM quercetin in the presence of 250 μM dexamethasone. ROS levels were assessed through staining with H2DCFDA, and the loss of ΔΨm was measured through DiOC(3)6 by flow cytometry. Values represent the mean ± SE (n = 8). Significant differences were determined using Dunnett’s test (* *p* < 0.05, compared with the untreated group; # *p* < 0.05, compared with the dexamethasone-treated group).

The effects of quercetin on ROS production and apoptosis in dexamethasone-treated C2C12 myotube cells are shown in Figure 3. ROS levels significantly increased in the dexamethasone-treated C2C12 myotube cells (*p* < 0.05; Figure 3A). Moreover, ROS production significantly decreased in a concentration-dependent manner after cotreatment of dexamethasone-treated C2C12 myotube cells with quercetin. Quercetin exhibited a similar trend of decrease in apoptosis induced by dexamethasone (Figure 3B). The results indicated that quercetin significantly reduced ROS production and apoptosis in dexamethasone-treated C2C12 myotube cells.

### 2.4. Effects of Dexamethasone on Changes in Apoptosis-Regulated Protein Levels in C2C12 Myotube Cells

**Figure 4:**
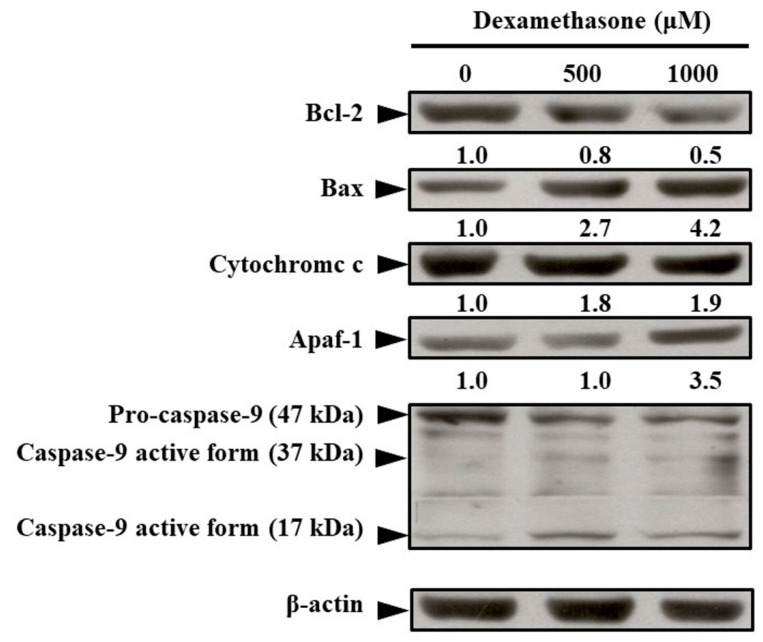
Effect of dexamethasone on apoptotic signaling of C2C12 myotube cells. The cells were treated with or without 500 and 1000 μM dexamethasone for 24 h. Thereafter, cell lysates were collected and blotted using specific antibodies, including Bcl-2, Bax, cytochrome c, Apaf-1, pro-caspase-9, and caspase-9, and then subjected to Western blot analysis as described in the Materials and Methods section. Each lane of protein signaling was normalized to β-actin. Each band was quantified using ImageJ software.

To demonstrate apoptosis-regulated protein expression in dexamethasone-treated C2C12 myotube cells, Western blotting analysis was performed. As shown in Figure 4, dexamethasone reduced the expression of Bcl-2 protein. Subsequently, dexamethasone increased the expression of apoptosis-related proteins, such as Bax, cytochrome C, Apaf-1, and mitochondrial-related apoptotic proteins (Figure 4).

### 2.5. Effects of Quercetin on Molecular Level Changes in Apoptosis-Regulated Protein and Caspase-3/9 Activities in Dexamethasone-Treated C2C12 Myotube Cells

**Figure 5:**
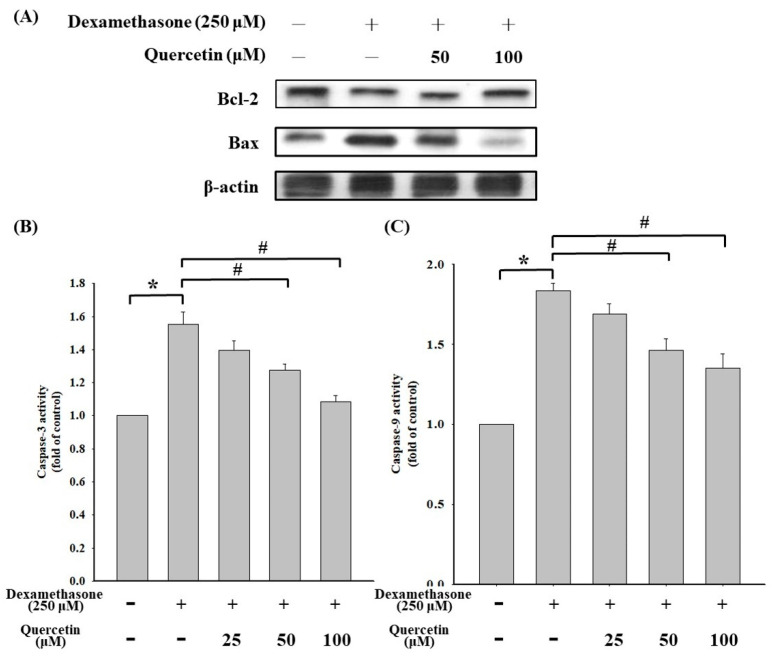
(**A**) Effect of quercetin on apoptotic signaling protein in dexamethasone-treated C2C12 myotube cells. The cells were cotreated with or without 50 and 100 μM quercetin in the presence of 250 μM dexamethasone for 24 h. Cell lysates were collected and blotted using specific antibodies, including Bcl-2 and Bax, and then subjected to Western blot analysis as described in the Materials and Methods section. Each lane of protein signaling was normalized to β-actin. (**B**) Effect of quercetin on caspase-3 protein in dexamethasone-treated C2C12 myotube cells. The cells were cotreated with or without 25, 50, and 100 μM quercetin in the presence of 250 μM dexamethasone for 24 h (**C**) Effect of quercetin on caspase-9 protein in dexamethasone-treated C2C12 myotube cells. The cells were cotreated with or without 25, 50, and 100 μM quercetin in the presence of 250 μM dexamethasone for 24 h ad then subjected to Western blot analysis as described in the Materials and Methods section. Each band was quantified using ImageJ software. Values represent the mean ± SD (n = 3). Significant differences were determined using Dunnett’s test (* *p* < 0.05, compared with the untreated group; # *p* < 0.05, compared with the dexamethasone-treated group).

Western blotting analysis was performed to examine the expression of Bcl-2/Bax in dexamethasone-treated C2C12 myotube cells, and the results are shown in Figure 5. Quercetin at concentrations of 50 and 100 μM significantly increased the expression of Bcl-2 protein compared with that in dexamethasone-treated cells (Figure 5A). Quercetin at concentrations of 25, 50, and 100 μM significantly reduced caspase-3 activity compared with that in dexamethasone-treated cells (Figure 5B). In addition, quercetin at concentrations of 25, 50, and 100 μM significantly reduced caspase-9 activity compared with that in dexamethasone-treated cells (Figure 5C). The results indicated that quercetin could upregulate Bcl-2 expression and reduce the activities of caspase-3/9 in dexamethasone-treated C2C12 myotube cells.

## 3. Discussion

Glucocorticoids are agents of DNA damage and repair and may have influenced oncogenic transformation in the present study [43,44]. Glucocorticoids can increase the production of ROS and reactive nitrogen species (RNS), which are capable of inducing DNA damage by causing a breakage of DNA strands and DNA base pair changes, leading to muscle wasting [45,46,47]. As seen from results in Figure 1 and Figure 3, dexamethasone reduced cell viability via increasing of ROS products. Subsequently, dexamethasone increased the expression of apoptosis-related proteins, such as Bax, cytochrome C, Apaf-1, and mitochondrial-related apoptotic proteins (Figure 4). Increasing evidence suggests that increased ROS and RNS levels reduce enzymatic antioxidant protection because of the oxidative stress generated from the peroxidative factors in pathophysiological mechanisms, leading to sarcopenia [48]. ROS production involves various pathways, mostly those relevant to the mitochondrial electron transport chain, wherein the transfer of a single electron to molecular oxygen results in a monovalent reduction of oxygen, which leads to the formation of superoxide ions. Superoxide ions may also be formed by enzymatic NADPH oxidase enzymes or the xanthine/xanthine oxidase system. NADPH oxidase was first discovered in phagocytes and was found to induce cell apoptosis [49]. Quercetin is a potent ROS scavenger, and its antioxidant capacity is due to the presence of two pharmacophores within the optimal configuration for free radical scavenging in its molecule, namely, the catechol group in the B ring and the OH group at position 3 [50].

**Figure 6:**
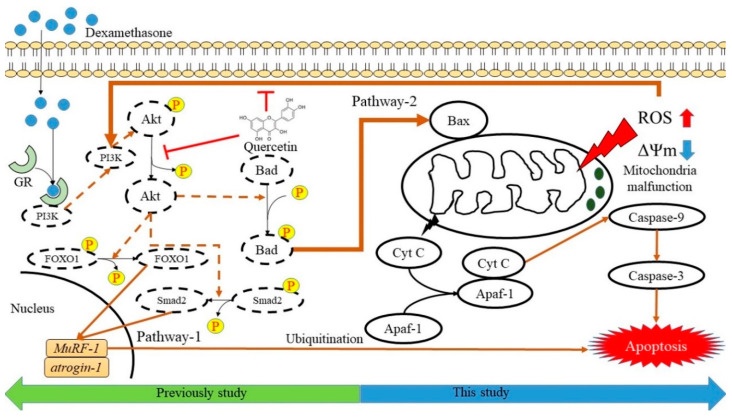
Possible antioxidant signaling pathways regulating dexamethasone-induced muscle apoptosis by quercetin. Pathway-1 was described in previous studies as the PI3K/Akt pathway [12], and pathway-2 was demonstrated in our study to regulate mitochondrial caspase-dependent apoptosis.

We examined the relationship between dexamethasone-induced apoptosis and Bax/Bak-mediated mitochondrial outer membrane permeabilization (MOMP) regulation in C2C12 myotube cells (Figure 6). In addition, oligomerization of the Bcl-2 family of proteins, Bax/Bak, is an irreversible step leading to caspase-dependent apoptosis or caspase-independent cell death in pathway-2 [51,52]. Dexamethasone induces glucocorticoid receptor (GR) expression, which in turn induces RNS generation [53]. This reaction causes almost immediate nongenomic actions on other signaling processes as a result of internal proteins dissociating from the GR complex stimulation [53,54,55]. However, soluble proteins that regulate MOMP are released from the mitochondrial intermembrane space into the cytoplasm. Cytochrome C binds to monomeric Apaf-1, leading to a conformational change and oligomerization. Procaspase-9 is recruited by heptameric Apaf-1 complexes to form apoptosomes. In this study, dexamethasone-induced muscle apoptosis was induced through the activation of executioner caspases-9 and -3 (Figure 6). Cell apoptosis occurs through a gradual loss of mitochondrial function and/or release of mitochondrial proteins that causes cell death in a caspase-independent manner [56]. Mitochondrial malfunction symbolized the induction of apoptosis and played a key role in the apoptosis of skeletal muscle cells. In addition to excessive local ROS generation caused by sublethal mitochondrial injury, mitochondrial malfunction leads to FoxO and NF-κB. Muscle atrophy signals protein activity to sustain protein breakdown because the oxide pressure trigging those apoptotic and inflammatory protein overexpression [57,58,59,60]. The metabolites of quercetin have been suggested to modulate the cell’s own antioxidant defense mechanisms [61,62], indicating that quercetin may act as a pro-oxidant rather than an antioxidant [62,63]. Oxidative stress may indeed increase the cell’s own antioxidant defenses, thereby protecting the cells [64,65]. The possible antioxidant signaling pathways by which quercetin regulates dexamethasone-induced muscle apoptosis are summarized in Figure 6. In pathway-1, studies have reported that glucocorticoids can induce ROS or RNS production and DNA damage through an iNOS-mediated pathway [53], indicating that pathological conditions associated with muscle loss were characterized by the upregulation of atrogin-1, MuRF-1, and many E3 ubiquitin ligases in skeletal muscle [66,67,68,69,70,71] and the upregulation of myostatin [11]. Dexamethasone not only upregulates atrogin-1 and MuRF-1 but also promotes myostatin. The myostatin gene activates Smad2 signaling and inhibits Akt activation, thereby altering the expression of multiple genes involved in the regulation of protein degradation and increasing skeletal muscle apoptosis [9,72,73]. Previous studies on ROS-related factors inducing myoblast apoptosis have shown that the p53 and Akt pathways are involved in cell apoptosis and ROS accumulation [74,75,76].

Our results revealed that quercetin protects against dexamethasone-induced apoptosis via two major pathways. First, quercetin reduces Akt dephosphorylation by reducing the expression of atrogin-1, which regulates atrogenes through the Akt-Foxo1 pathway [12]. Second, quercetin exhibits antioxidant properties and free radical scavenging capacities, which reverses mitochondrial ΔΨm imbalance. Mitochondria are the resource of cellular energy, and their malfunction could induce insufficient energy supply and activate of several intracellular signaling pathways such as the AMPK pathway, autophagy, and/or apoptosis [35,77,78]. In addition, quercetin significantly reduced caspase-9 activity compared with that in dexamethasone-treated cells (Figure 5C). The results indicate that quercetin could upregulate Bcl-2 expression and reduce the activities of caspase-3/9 in dexamethasone-treated C2C12 myotube cells. Phenolic compounds such as quercetin, rutin, caffeic acid, curcumin, and resveratrol have been investigated for their abilities to scavenge superoxide anion radicals generated in isolated heart mitochondria [79,80]. Because of the presence of hydroxyl groups and conjugated π-orbitals, these antioxidant phenolic compounds can effectively remove O2^•−^ formed in mitochondria [79]. Furthermore, antioxidants such as quercetin, resveratrol, sulforaphane, and glabridin prevent dexamethasone-induced muscle atrophy through the regulation of the Akt/Foxo1 axis in C2C12 myotubes [12,32,33,81]. In addition to the Akt pathway, our results demonstrated that quercetin protects dexamethasone-induced muscle atrophy by regulating Bax/Bcl-2 protein expression and reversing ΔΨm imbalance, thereby suppressing apoptosis.

## 4. Materials and Methods

### 4.1. Materials

Quercetin, dexamethasone, thiazolyl blue tetrazolium bromide (MTT), the In Situ Cell Death Detection Kit (Fluorescein), and other chemicals and reagents were purchased from Sigma–Aldrich, Merck KGaA (Darmstadt, Germany), unless otherwise stated. All primary antibodies and anti-mouse and antirabbit immunoglobulin (Ig) G horseradish peroxidase (HRP)-linked secondary antibodies were purchased from GeneTex (Hsinchu, Taiwan). Z-VAD-FMK (a pan-caspase inhibitor) and Muse Caspase-3/9 assay kits were obtained from Millipore, Merck KGaA (Darmstadt, Germany). 2′, 7′-Dichlorodihydrofluorescein diacetate (H2DCFDA) and 3, 3′-dihexyloxacarbocyanine iodide [DiOC6(3)] were obtained from Molecular Probes, Thermo Fisher Scientific (Waltham, MA, USA). Dulbecco’s modified Eagle’s medium (DMEM) was purchased from Sigma–Aldrich (Lenexa, KS, USA). Fetal bovine serum (FBS), L-glutamine, penicillin/streptomycin, and trypsin-EDTA were purchased from HyClone, GE Healthcare Life Sciences (Logan, UT, USA).

### 4.2. Cell Culture

Murine myoblast C2C12 cell line was purchased from the American Type Culture Collection (Manassas, VA, USA). C2C12 cells (2–4 passages) were cultured in DMEM supplemented with 10% FBS until 100% confluence at 37 °C in a humidified 5% CO_2_ atmosphere. To attain confluency, proliferating myoblasts were induced to differentiate into myotubes by replacing the culture medium with DMEM supplemented with 2% horse serum (Sigma–Aldrich, Lenexa, KS, USA) for 6 days. The cultural medium was renewed every 2–3 days. After the myotubes were fully differentiated, the cells were treated with 25, 50, 75, and 100 μM of quercetin for 24 h. Subsequently, the medium was cotreated with 250 μM dexamethasone to induce atrophy for 4 h. Cells were then harvested and subjected to the cell viability test, ROS production assay, transferase-mediated d-UTP nick end labeling (TUNEL) assay, caspase-3/9 assay, and Western blotting analysis.

### 4.3. Cytotoxicity Assay

The effect of dexamethasone and quercetin cotreatment was detected using an MTT assay by following previously described instructions [82,83]. The differentiated C2C12 myotube cells (1 × 10^4^ cells/well) were cultured onto 96-well plates and then exposed to various concentrations (25, 50, 75, and 100 μM) of quercetin for 24 h. After quercetin treatment, cells were further exposed to 250 μM dexamethasone for 4 h to induce atrophy. Subsequently, 10 μL of the MTT solution (5 mg/mL) was added to each well followed by a 3 h incubation. After the medium was removed, the purple formazan crystals formed were solubilized with 100 μL dimethyl sulfoxide (DMSO). The absorbance of the formazan crystals in the lysate was measured using a microplate reader at 570 nm as previously described [84].

### 4.4. Morphological Assay

Cells (1 × 10^5^ cells/well) were plated onto 12-well plates and then treated with or without 100 μM quercetin for 24 h. After quercetin treatment, the cells were cotreated with 250 μM dexamethasone for 4 h to induce atrophy. The cells were subsequently observed and photographed using a phase-contrast microscope at a magnification of ×200.

### 4.5. Apoptosis Analysis through TUNEL Assay

The cells (1 × 10^5^ cells/mL) were cultured in various concentrations (25, 50, and 100 μM) of quercetin for 24 h. After quercetin treatment, the cells were cotreated with 250 μM dexamethasone for 4 h to induce atrophy. The cells were then washed with phosphate buffered saline and harvested subsequently. Flow cytometry (BD FACSCalibur Flow Cytometer, BD Biosciences, San Jose, CA, USA) was conducted to detect apoptotic situation, and the cells were stained using the In Situ Cell Death Detection Kit (Fluorescein; Sigma–Aldrich, Merck KGaA, Mannheim, Germany) according to the manufacturer’s instructions. The terminal deoxynucleotidyl TUNEL positive cells were quantified using the BD Cell Quest Pro Software version 5.1 (BD Biosciences) as previously described [84].

### 4.6. Caspase-3/9 Assay and Their Specific Inhibitor Activities

The cells (1 × 10^4^ cells/well) were cultured onto 96-well plates and treated with 500 μM dexamethasone for 24 h before pretreatment with or without 10 μM Z-VAD-FMK (a pan-caspase inhibitor) for 1 h; they were then treated with 0.5 mg/mL MTT solution for 2 h. Finally, 100 μL DMSO was added onto each well to replace the culture medium to dissolve formazan crystals.

To measure caspase protein expression, the cells (1 × 10^5^ cells/mL) were cultured in quercetin at various concentrations (25, 50, and 100 μM) for 24 h. The cells were then cotreated with 250 μM dexamethasone to induce atrophy for 4 h. Subsequently, cells were cultured with a working solution of the Muse Caspase-3/9 Assay Kit (Millipore; Merck KGaA) and then harvested though centrifugation at 400× *g* according to the manufacturer’s protocol (Caspase-3 and Caspase-9 Colorimetric Assay Kits, R&D System Inc., Minneapolis, MN, USA).

### 4.7. Western Blotting Analysis

The cells (1 × 10^5^ cells/mL) were cultured in 50 and 100 μM quercetin for 24 h. After quercetin treatment, the cells were cotreated with 250 μM dexamethasone for 4 h to induce atrophy. Finally, the cells were lysed with Trident RIPA lysis buffer (GeneTex) to analyze the total protein concentration during the exposure period. The protein concentration was detected using the Pierce BCA Protein Assay Kit (Thermo Fisher Scientific). Protein samples (40 μg) from each well were loaded on a 10%–12% sodium dodecyl sulfate–polyacrylamide gel and transferred through electroblotting to the Immobilon-P Transfer membrane (Merck KGaA) for 1 h. The membrane was blocked with 5% skim milk in tris-buffered saline with 0.1% Tween 20 (TBST) and then incubated with primary antibodies [Bcl-2, Bax, and β-actin (1:5,000 dilution)] at 4 °C overnight. On the following day, the membrane was washed with TBST and hybridized with the appropriate antirabbit (cat. no. GTX213110-01) and anti-mouse (cat. no. GTX213111-01) IgG HRP-linked antibodies (1:10,000 dilution) for 1 h at room temperature. An enhanced chemiluminescence kit (Immobilon Western Chemiluminescent HRP substrate, Merck Millipore) was used to visualize protein bands, and protein band quantification was measured using NIH ImageJ software (version 1.47), as previously described [84].

### 4.8. Determination of ROS Levels through Flow Cytometry

Cells (1 × 10^5^ cells/mL) were cultured in the presence of various concentrations (25, 50, and 100 μM) of quercetin for 24 h. After quercetin treatment, the cells were cotreated with 250 μM dexamethasone for 4 h to induce atrophy. The cells were then harvested through centrifugation at 400× *g* for 5 min, and the cell pellets were suspended in 500 μL H2DCF-DA (an ROS indicator dye, 10 μM) staining solution at 37 °C for 30 min. The ROS levels in the cells were then determined using flow cytometry, as previously described [85,86].

### 4.9. Detection of Mitochondrial Electrical Potential (ΔΨm)

C2C12 cells were cultured in various concentrations (125, 250, 500, and 1000 μM) of dexamethasone for 24 h. Further, the treated cells were harvested and labeled with 500 nM DiOC6(3) at 37 °C for 30 min. The fluorescence intensity corresponding to ΔΨm was analyzed through flow cytometry, as previously described [84,87].

### 4.10. Statistical Analysis

All results are presented as the mean ± standard deviation (SD) of triplicate data. The data were statistically analyzed with one-way analysis of variance, followed by Dunnett’s test using SPSS software version 16.0 (SPSS, Chicago, IL, USA). A *p* value of < 0.05 was considered statistically significant.

## 5. Conclusions

This study demonstrated that quercetin can reduce dexamethasone-induced mitochondrial malfunction in skeletal muscle cells through the downregulation of Bax and ROS and the reversal of the ΔΨm imbalance. As a food supplement, quercetin has substantial benefits in relieving dexamethasone-induced skeletal muscle injury and could serve as an alternative natural plant resource for the prevention or treatment of muscle atrophy syndrome. The potent antioxidant activity of quercetin renders it suitable to be used as a dietary supplement for patients under long-term dexamethasone treatment to regulate mitochondria health in skeletal muscle.

## Abbreviations

Cyt C	Cytochrome C
GR	Glucocorticoid receptors
iNOS	Inducible nitric oxide synthase
MOMP	Mitochondrial outer membrane permeabilization
NADPH	Nicotinamide adenine dinucleotide phosphate
ROS	Reactive oxygen species
RNS	Reactive nitrogen species
ΔΨm	Mitochondrial Membrane Potential
